# Evaporation and Electrowetting of Sessile Droplets on Slippery Liquid-Like Surfaces and Slippery Liquid-Infused Porous Surfaces (SLIPS)

**DOI:** 10.1021/acs.langmuir.0c02020

**Published:** 2020-09-03

**Authors:** S. Armstrong, G. McHale, R. Ledesma-Aguilar, G. G. Wells

**Affiliations:** † Smart Materials & Surfaces Laboratory, Faculty of Engineering & Environment, Northumbria University, Newcastle upon Tyne, NE1 8ST, U.K.; ‡ School of Engineering, University of Edinburgh, Sanderson Building, Edinburgh, EH9 3FB, U.K.

## Abstract

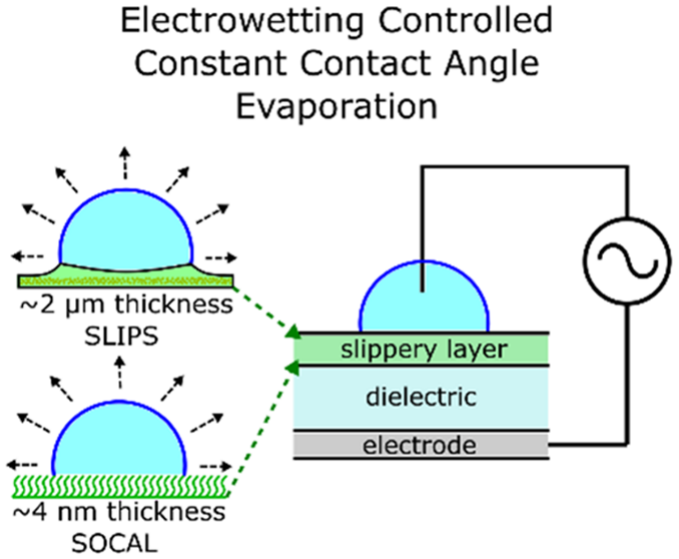

Sessile droplet evaporation underpins a wide range of applications from inkjet printing to coating. However, drying times can be variable and contact-line pinning often leads to undesirable effects, such as ring stain formation. Here, we show voltage programmable control of contact angles during evaporation on two pinning-free surfaces. We use an electrowetting-on-dielectric approach and Slippery Liquid-Infused Porous (SLIP) and Slippery Omniphobic Covalently Attached Liquid-Like (SOCAL) surfaces to achieve a constant contact angle mode of evaporation. We report evaporation sequences and droplet lifetimes across a broad range of contact angles from 105°–67°. The values of the contact angles during evaporation are consistent with expectations from electrowetting and the Young-Lippman equation. The droplet contact areas reduce linearly in time, and this provides estimates of diffusion coefficients close to the expected literature value. We further find that the total time of evaporation over the broad contact angle range studied is only weakly dependent on the value of the contact angle. We conclude that on these types of slippery surfaces, droplet lifetimes can be predicted and controlled by the droplet’s volume and physical properties (density, diffusion coefficient, and vapor concentration difference to the vapor phase) largely independent of the precise value of contact angle. These results are relevant to applications, such as printing, spraying, coating, and other processes, where controlling droplet evaporation and drying is important.

## Introduction

The evaporation of sessile droplets of liquids from solids occurs in many applications including heat exchange,^1^ particle deposition,^2^ and inkjet printing.^3^ Due to its importance to a wide range of physical processes, the literature is extensive (see, e.g., Erbil,^4^ Cazabat and Guéna,^5^ and Larson^6^). For a sessile droplet, the presence of the solid surface results in an evaporation rate, and hence drop drying time, which depends on the droplet’s contact angle. Inhomogeneities in practical surfaces also mean there is contact line pinning which has consequences for predicting and controlling evaporation. It can prevent the contact area between the liquid and solid with it being circular thus giving irregular drying spots and drying times. If the evaporating droplet is a suspension, it can cause nonuniform particle deposition, similar to the coffee-ring stain effect.^6^ This can cause problems in a broad range of applications from nonuniform delivery of the active components in aerosols used in pesticides to nonuniform fluorescence in spotted microarrays.^2,7−9^ One way to prevent ring-stain patterns is to remove contact line pinning so that the contact line is completely mobile during evaporation, but this is the exception on solid surfaces unless active means, such surface acoustic wave^10^ or electrowetting-induced agitation of the liquid, are used.^11^ It is therefore desirable to investigate contact angle dependence of evaporation of droplets on surfaces which do not have contact line pinning to understand control of the evaporation sequence and droplet lifetimes.

One possible approach to removing contact line pinning is to use superhydrophobic surfaces with contact angles above ca. 150°,^12−14^ and the first example of using such a surface for evaporation was provided by McHale et al.^15^ In their case, the texture of their micropost surface led to quantization of the receding contact line into stepwise jumps from pillar to pillar before a collapse into the structure and complete pinning. In other cases, evaporation of sessile droplets from nanoparticle-based superhydrophobic surfaces has shown droplets evaporate for a relatively constant contact angle ca. 150°.^16^ However, these surfaces have high contact angles toward 180° with small contact areas to create a Cassie–Baxter state and so use texture or roughness for which there remains the risk of impalement of the drop into the texture through, for example, pressure-induced or condensation of vapor-induced transitions, to the Wenzel state. An alternative approach to removing contact line-pinning, which avoids the risk of an impalement transition, is the use of a Slippery Liquid-Infused Porous Surface (SLIPS),^17^ and this has been shown to support a constant contact angle type mode of evaporation.^18^ However, while SLIPS provide a smooth surface, the droplet is never in direct contact with the underlying solid but rests on the layer of lubricant used to infuse the porous (or textured) solid surface structure. Thus, the observed contact angle is an apparent contact angle and is not determined by interaction with the underlying solid surface, and the lubricant may alter the evaporation rate. This apparent contact angle on SLIPS can be theoretically described,^19^ and for thin layers of lubricant, the contact angle can be predicted using a liquid/lubricant form of Young’s law, which also provides an upper bound for thicker layers of lubricant.^19,20^ Most recently, Wang and McCarthy introduced a new type of slippery surface which they named Slippery Omniphobic Covalently Attached Liquid (SOCAL) surfaces obtained through acid-catalyzed graft polycondensation of dimethyldimethoxysilane.^21^ This has allowed the observation of pinning-free constant contact angle mode evaporation on smooth slippery liquid-like, but solid, surfaces.^22^ In the case of SLIPS, created using silicone oil as a lubricant, and for SOCAL, the contact angle for droplets of water are ca. 108° and 104° and so can be regarded as hydrophobic surfaces. At present, there are no examples of pinning-free evaporations of sessile water droplets from hydrophilic surfaces.

An outstanding challenge for studies of pinning-free evaporation of sessile droplets is control of the range of contact angles on smooth slippery surfaces. Here, our primary objective is to address this challenge by introducing electrowetting-on-dielectric (EWOD)^23,24^ as a technique to control the contact angle during evaporation. Electrowetting is an important tool that can manipulate and control droplets, e.g., in microfluidics,^25−27^ liquid lenses,^28^ and optofluidics,^29^ and can be used with SLIP surfaces (see e.g., ref (30−32)). In this type of electrowetting, the solid–liquid contact area of a sessile droplet acts as one electrode in a capacitive structure allowing the contact angle to be reduced by the application of a voltage. Electrowetting does not alter the spherical cap shape of small sessile droplets provided the voltage is below the saturation voltage,^33^ and since charges are stored at the solid–liquid interface, we anticipate it will not significantly influence the evaporation of sessile droplets. In the remainder of this paper, we describe the theory for the constant contact angle mode of evaporation and the creation of two types of slippery surfaces (SOCAL and SLIPS) in an electrowetting configuration. We then report data for the constant contact angle evaporation mode over a range of contact angles from 105° to 67° including a comparison to expected values from the Young-Lippmann equation to show consistency with theory and provide confidence in the technique. We report estimates for the diffusion coefficient of water vapor and for droplet lifetimes and show that droplet lifetime is largely insensitive to the precise value of the contact angle over the range studied.

## Theory of Constant Contact Angle Mode Evaporation

Picknett and Bexon provided the first solution for the diffusion-controlled evaporation of a spherical cap shaped sessile droplet on a smooth and homogeneous surface and identified two ideal modes.^34^ In the first mode, the contact line is completely pinned, and the evaporation occurs with a constant contact radius, so that the contact angle decreases throughout the entire evaporation. This mode of evaporation has been achieved experimentally and is widely studied.^4,35^ In the second mode the contact line is mobile, and the contact angle remains constant, resulting in a linear decrease in the contact area with time. Constant contact angle mode evaporation on surfaces has been experimentally difficult to observe because surfaces tend to exhibit contact angle hysteresis and contact line pinning. In practice, most droplets evaporate in a stick–slip mode of evaporation, where the contact line is repeatedly pinned on the surface, depinning when the contact angle is sufficiently out of equilibrium to exceed the force necessary to move the contact line. A number of authors have also observed another mode of evaporation known as stick-slide mode evaporation, where the contact area and contact angle decrease at the same time, e.g., ref (34). In particular, Stauber et al. have provided a model to predict the lifetime of droplets in stick-slide, constant contact radius, and constant contact angle mode evaporation.^36,37^

In the ideal case without contact line pinning and when a droplet is in thermodynamic equilibrium, the contact angle, θ_*e*_, a sessile droplet makes with a smooth solid surface is determined by three interfacial tensions as described by Young’s law
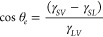
1where γ_*SV*_ is the solid–vapor interfacial tension, γ_*SL*_ is the solid–liquid interfacial tension, and γ_*LV*_ is the liquid–vapor interfacial tension.^38^ This applies to droplets in equilibrium with contact angles from 0°, where a droplet just forms a film between the solid surface and the surrounding vapor phase, to 180° where a droplet completely balls up on the surface.

For a small sessile droplet with a size below the capillary length *l*_*c*_ = (γ_*LV*_/ρg)^1/2^ where ρ is the density of the liquid and g = 9.81 ms^–2^ is the acceleration due to gravity, the droplet adopts an axially symmetric spherical cap shape with well-defined geometric parameters that can be measured from side profile images. These include the spherical cap radius *R*, contact radius *r*, contact angle θ, and the apex height *h*, above the contact surface. Geometrically, the volume, Ω, is defined as
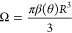
2where
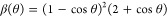
3and the contact radius is related to the spherical radius by *r* = *R*sin θ. In general, the rate for diffusion-limited loss of a liquid volume by evaporation through a liquid–vapor interface using a surface integral of the concentration gradient is
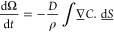
4where *D* is the diffusion coefficient of the vapor.^5^ Combining the geometrical assumptions with eq 4 and a concentration gradient model gives
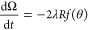
5where
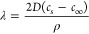
6Here, (*c*_*s*_ – *c*_*∞*_) is the difference in the vapor concentration at the liquid–vapor interface of the droplet *c*_*s*_, which is assumed to be its saturation value and that far removed from the droplet surface *c*_*∞*_, which is assumed to be its ambient value. For analyzing data, an exact solution for eq 5 was derived by Picknett and Bexon^34^ and they gave a numerically accurate polynomial interpolation, *f*_*PB*_(θ), for the exact solution *f*(θ) covering the entire contact angle range
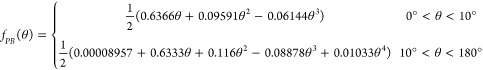
7where θ in the series is in radians. For the constant contact angle evaporation mode, the rate of change of the contact area is
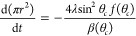
8where θ_*c*_ is the constant value for the contact angle. Thus, the contact area has a linear change with time from its initial value determined by the initial contact radius, *r*_*i*_, at *t* = 0, i.e.,
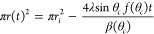
9Similarly, the rate of change in volume can be expressed in terms of the instantaneous volume and the constant contact angle
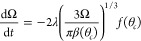
10and this integrates to give a 2/3 power law for the volume
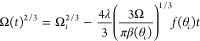
11where Ω_*i*_ is the initial droplet volume at *t* = 0. The droplet lifetime, *t*_*f*_, is then defined by the time at which the droplet contact area (eq 9), or equivalently the droplet volume (eq 11), vanishes, i.e.,
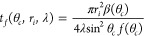
12which can also be written as
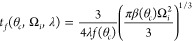
13Thus, the total droplet lifetime during constant contact angle evaporation of a spherical cap shaped sessile droplet depends on the value of the contact angle and the initial contact radius (or volume) and a parameter, λ, combining the diffusion coefficient, density of liquid, and the vapor concentration difference.

## Experimental Methods and Materials

**Figure 1:**
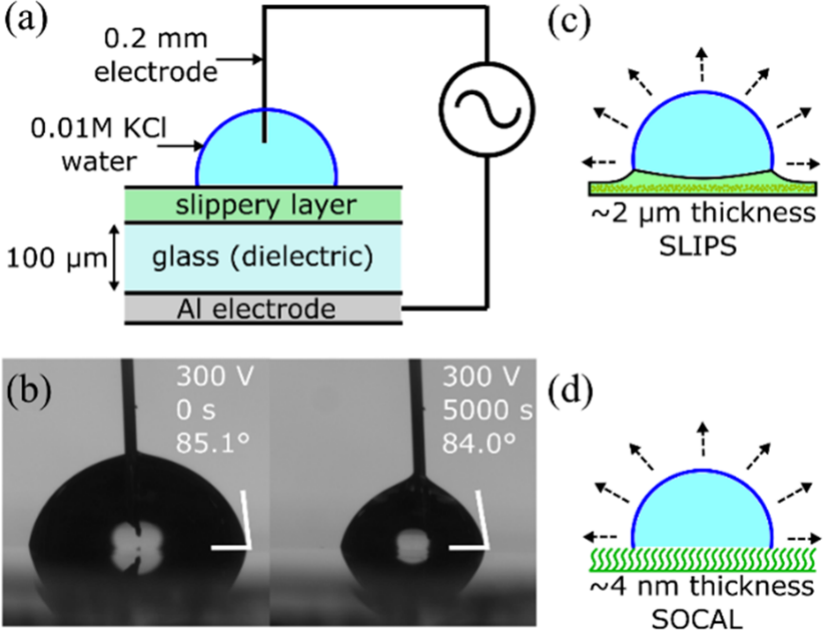
Electrowetting and evaporation on slippery surfaces: (a) Schematic of droplet in an electrowetting setup with a glass dielectric substrate and a slippery top layer. (b) Example images of droplets evaporating under fixed rms voltage (300 V) at constant angle on SOCAL. Sketches of the two types of slippery top-layers (c) Droplet on hydrophobic nanoparticle SLIPS, and (d) droplet on SOCAL.

Our experiments required surfaces that were both free of contact line pinning and had contact angles that could be adjusted to different constant values. To do this, we used two types of surfaces, SOCAL and SLIPS, as slippery layers on a glass substrate (as the dielectric) in an electrowetting configuration as described below (Figure 1). The electrowetting configuration allows the initial contact angle determined by the droplet-solid, droplet-lubricant, and other interfacial tensions to be reduced in a programmable manner by application of a voltage.

The electrowetting configuration to investigate evaporation at different constant contact angles on these surfaces is shown in Figure 1a with example evaporation images in Figure 1b and the schematics showing the two types of slippery surfaces in Figure 1c,d. An alternating current (AC) system was generated using a signal generator (TTi Instruments TGA1244) to generate a 10 kHz sinusoidal wave which was then amplified (Trek PZD700A) as a programmable root-mean-square (rms) voltage, *V*, within the range 0 to 450 V. The amplified signal was then applied to an aluminum-coated glass slide (100 μm, vapor deposited) as one electrode and a thin metal, 0.2 mm diameter, in the center of the droplet, as the second electrode. The cross sectional area of the needle is ∼0.13 mm^2^, compared to the surface area of a 8 μL droplet with 105° contact angle which is ∼15.6 mm^2^ (less than 1% of the total surface area); the needle is therefore not expected to have a significant effect on the spherical cap shape during the evaporation. The 100 μm thick glass coverslip on which the slippery coating (SOCAL or SLIPS) was attached acts as a dielectric that enables storage of capacitive energy and allows the contact area, and hence contact angle, to be adjusted by altering the balance between capacitive and interfacial energies. The droplets of deionized water used in the experiments had a volume of 8 μL, and 0.01 M KCl was added to ensure the electrical conductivity required for electrowetting. The thin metal electrode was lowered into the center of the droplet after deposition, and evaporation experiments were conducted at room temperature (22 ± 2 °C) at a controlled relative humidity of 70% within a transparent chamber to regulate the conditions local to the droplet. The chamber also shields the droplet from the presence of air drafts which might otherwise entrain the lubricant from a SLIP surface over an evaporating droplet.^39^ Droplet evaporation sequences were recorded using a camera at 0.05 frames per second, and contact radius *r* and contact angle θ measurements were determined using open-source pyDSA software.^40^ Experimentally, profiles of the droplet were accurately described by a spherical cap to within a slight distortion around the electrode needle. The volume of the droplet during evaporation was calculated using the contact radius and contact angle. The data set presented in the Results and Discussion is a representative sample of wider body of experiments, and each evaporation at a fixed voltage for each surface is the average of three evaporation sequences.

The first type of slippery surface used SLIPS samples prepared by taking new glass coverslips (Thorlabs, CG00K1) of thickness (100 ± 5) μm, coating them with 5 layers of Glaco Mirror Coat (Nippon Shine) to create a nanoparticle-based superhydrophobic porous structure, and then infusing a layer of lubricant by withdrawal from a bath of 20 cSt silicone oil (Sigma-Aldrich, 378348) at 0.1 mm s^–1^. Excess oil was rinsed off to ensure only a thin conformal oil layer remained on the surface so that there was no visible wetting ridge of oil on subsequent sessile droplets. The water contact angle hysteresis Δθ was determined by measuring advancing contact angle θ_*A*_ and receding contact angle θ_*R*_ through the average of three droplet inflation/deflation experiments in different locations on the substrate (Δθ = 0.4 ± 0.3°, θ_*A*_/θ_*R*_ = 109.6°/109.2°). Sliding angles α were also measured by placing a 20 μL droplet of deionized water on the surface and tilting the substrate until the droplet begins to slide, and the average of three measurements gives α_20*μL*_ = 0.2 ± 0.2°. The measured contact angle is consistent with theoretical expectations of ca. 108° based on a liquid-form of Young’s law (eq 4 in reference^20^) using an effective droplet-vapor interfacial tension as sum of the droplet-oil and oil-vapor interfacial tensions and indicates silicone oil should cloak the droplet-vapor interface despite the absence of a visible wetting ridge at the contact line.^19,20^

The second type of slippery surface used smooth liquid-like SOCAL surfaces prepared on glass samples (see refs (21 and 22)). New glass coverslips of thickness of (100 ± 5) μm were exposed to air plasma in a (Henniker HPT-100) at 30W for 20 min. The coverslips were then dipped in a reactive solution of 91.45 mL isopropyl alcohol (≥99.7%, Sigma-Aldrich, 292907), 8.16 mL dimethyldimethoxysilane (95%, Sigma-Aldrich, 104906), and 0.39 mL sulfuric acid (95.0–98.0%, Sigma-Aldrich, 258105) for 5–10 s and then slowly removed. These coated glass slides were subsequently placed in a bespoke humidity chamber for 20 min at 60% ± 2% relative humidity to allow the acid-catalyzed polycondensation to take place. After this time, the surface was rinsed with isopropyl alcohol, toluene (≥99.5%, Sigma-Aldrich, 179418), and deionized water (type III, purified in an Elga PURELAB Option-Q lab water purification system) to remove any remaining reactive solution. This creates flexible polydimethylsiloxane chains approximately 4 nm in length that allow mobility of the droplet contact-line thereby minimizing contact line pinning.^21^ For the confirmation of successful and homogeneous coating, contact angle hysteresis and sliding angles were measured in the same manner as that for the first surface and were determined to be Δθ = 1.0 ± 0.5°, θ_*A*_/θ_*R*_ = 105.7°/104.7°, and α_20*μL*_ = 5.6 ± 0.4°. These values are in good agreement with Wang and McCarthy who reported Δθ = 1.0°, θ_*A*_/θ_*R*_ = 104.6°/103.6°, and α_20*μL*_ = 4° for droplets of water. Using the same interfacial tensions as those for the silicone oil in the SLIPS but assuming the PDMS chains on a SOCAL surface cannot cloak the droplet-vapor interface, the liquid-form of Young’s law suggests the water droplet should have a contact angle on SOCAL of ca. 104°, and this is consistent with the measured value.

## Results and Discussion

### Constant Contact Angle Evaporation and the Diffusion Coefficient

**Figure 2:**
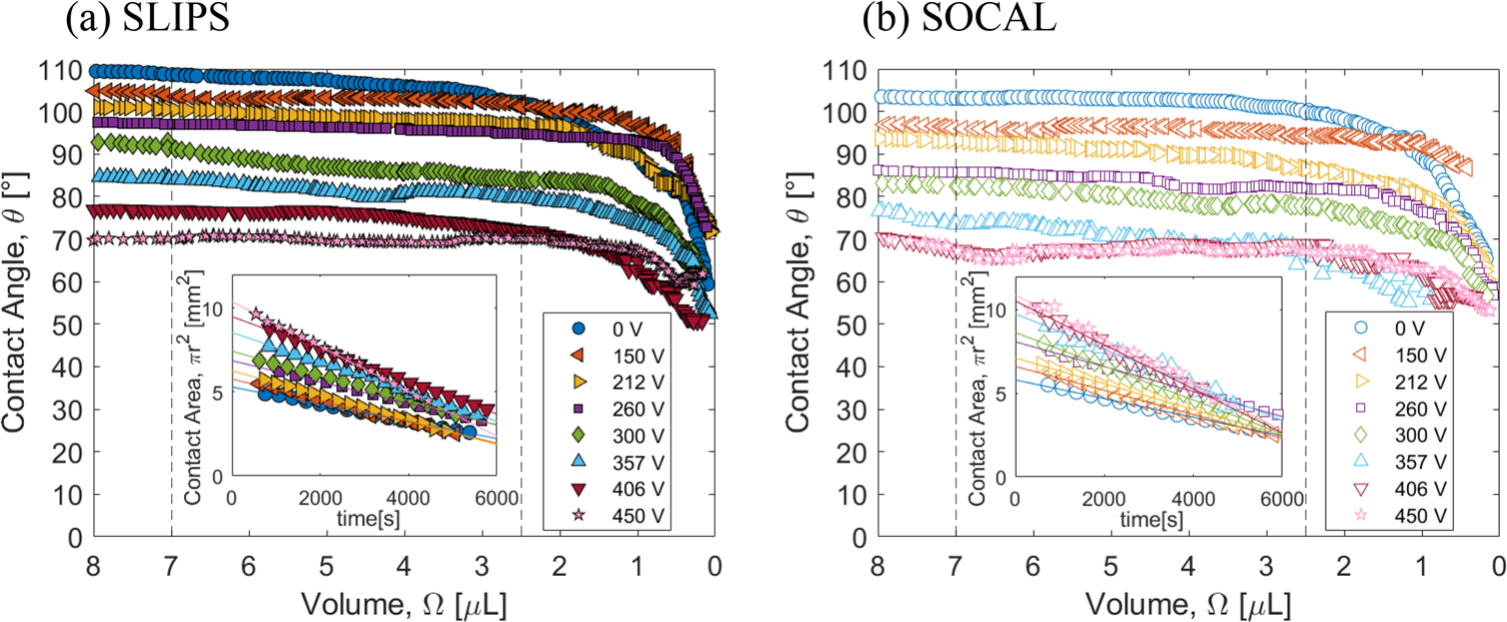
Contact angle as a function of reducing volume for 0.01 M KCl deionized water droplets evaporating at fixed electrowetting voltages on: (a) SLIPS and (b) SOCAL surface. Inset shows contact area as a function of time for the constant contact angle regime indicated by dashed lines.

We first discuss the qualitative features of the droplet evaporation. We observed that after a brief initial period (corresponding to a volume reduction from 8 to 7 μL), the contact angle remained approximately constant during the evaporation for most of the evaporation period on both types of slippery surfaces (Figure 2). In the last stage of the evaporation (corresponding to a volume reduction from 2.5 to 0 μL), we observe a decrease in the contact angle for both SLIPS and SOCAL surfaces. We rule out an effect from electric field causing the decrease in contact angle when the droplet is small, as the decrease also occurs for the 0 V evaporation. In our previous study on sessile water droplets evaporating on SOCAL surfaces, we suggest the decrease in contact angle could be due self-pinning after the precipitation of the trace salt in the droplet,^22^ and it is plausible such an effect is occurring in our current study. We now look in detail at the constant contact angle region of the evaporation. Focusing on SLIP surfaces, the droplet evaporation sequences were consistent with prior literature.^18^ However, because the samples here use thin conformal SLIP layers based on a hydrophobic nanoparticle coating, rather than lithographically produced micropillar textures with thicker layers of lubricant, there were no visible wetting ridges around the contact line. This is an improvement for evaporation studies since the oil in a wetting ridge removes some of the droplet-vapor surface area for evaporation. The application of a constant amplitude electrowetting voltage reduced the initial contact angle in a reproducible manner over many voltage cycles on the SLIP surfaces consistent with our previous report.^32^Figure 2a provides the first reports of droplet evaporation sequences with voltage selectable constant contact angle (ca. 70° to 105°) on SLIPS and covers the voltage range up to the saturation region well-known for electrowetting (see, e.g., ref (41)).

Focusing now on SOCAL surfaces, we observed the evaporation sequences to be consistent with that on the SLIPS surfaces and at zero applied voltage consistent with a prior report of evaporation on SOCAL.^41^ We were also able to reduce the contact angle by the application of the electrowetting voltage and observe, for the first time, evaporation sequences with voltage selectable constant contact angle (ca. 67° to 102°) on SOCAL (Figure 2b). When repeatedly cycling the electrowetting voltage the contact angle hysteresis increased to ca. 3.6 ± 0.4°, which nonetheless remains low compared to other hydrophobic coatings. In such experiments, the contact angle measured during the constant contact angle period of evaporation at zero voltage after the first cycle was reduced from (102.1 ± 1.2°) to (94.9 ± 1.5°). However, it was possible to apply a constant electrowetting voltage to a freshly deposited droplet on different areas of a SOCAL surface and observe smoothly receding contact lines as the droplets evaporated. Figure 2b shows such data with each data point of an average of three droplets on a sample and shows constant contact angles of 102.1 ± 1.2° to 67.2 ± 3.0° for voltages with rms values between 0 and 450 V. The corresponding contact areas of droplets decrease linearly in time during the constant contact angle period of the evaporation (Figure 2a inset).

We now consider the quantitative analysis of the constant contact angle regime for both types of slippery surfaces to confirm the absolute slopes from the data in Figure 2 are physically reasonable. Rearranging eq 9, the diffusion coefficient can be determined from the evaporation of a droplet using the average rate of change in contact area (slope in the insets to Figure 2), i.e.,
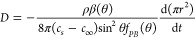
14where *f*(θ) is evaluated using the Picknett and Bexon interpolation formula (eq 7). Table 1 shows these calculated diffusion coefficients across the electrowetting voltage range (prior to contact angle saturation) are in good agreement with the literature diffusion coefficient. On the SLIP surfaces, the average diffusion coefficient measured experimentally is *D*_*Exp*_ = (2.06 ± 0.26) × 10^–5^ m^2^s^–1^ compared to the literature value of *D*_*lit*_ = (2.41 ± 0.05) × 10^–5^ m^2^s^–1^.^42^ On the SOCAL surfaces, the experimental average was found to be *D*_*Exp*_ = (2.14 ± 0.21) × 10^–5^ m^2^s^–1^, and using data for droplets on both types of surfaces, the experimental average was *D*_*Exp*_ = (2.10 ± 0.24) × 10^–5^ m^2^s^–1^. We also verified that the 2/3 power law for the drop volume (i.e., eq 11) was obeyed, and the slopes from that analysis are also given in Table 1 along with the value determined for the constant contact angle and the droplet lifetime (see analysis and discussion in the section Dependence of Initial Contact Angle on the Voltage). These results also support the assumption that electrowetting does not significantly alter the evaporation of sessile droplets from these surfaces.

**Table 1:** Experimentally Determined Diffusion Coefficients

**slippery**	**rms voltage**	**constant contact angle, θ**_**c**_	**d(π*r***^**2**^**)/d*t***	**dΩ**^**2/3**^**/d*t***	**total evap time, *t*_f_**	***D***_**Exp**_
**layer**	**[V]**	**[°]**	**[×10**^**-4**^**mm**^**2**^**s**^**-1**^**]**	**[×10**^**-3**^**μL s**^**-1**^**]**	**[s]**	**[×10**^**-5**^**m**^**2**^**s**^**-1**^**]**
**SLIPS**	0	105.3 ± 1.6	–5.18 ± 0.03	–4.49 ± 0.03	8034 ± 960	1.84 ± 0.06
	150	102.5 ± 2.6	–6.60 ± 0.09	–4.43 ± 0.05	7877 ± 940	2.17 ± 0.07
	212	98.2 ± 2.2	–7.31 ± 0.05	–4.97 ± 0.04	9416 ± 1100	2.28 ± 0.08
	260	95.8 ± 0.4	–6.42 ± 0.02	–3.98 ± 0.02	8698 ± 1040	1.89 ± 0.05
	300	86.3 ± 3.6	–7.15 ± 0.06	–4.25 ± 0.04	10876 ± 1300	1.76 ± 0.09
	357	81.0 ± 6.0	–8.70 ± 0.09	–4.35 ± 0.02	9255 ± 1100	1.95 ± 0.16
	406	74.6 ± 2.2	–9.83 ± 0.05	–4.46 ± 0.02	9004 ± 1070	2.03 ± 0.09
	450	70.1 ± 1.4	–13.27 ± 0.12	–5.12 ± 0.05	10151 ± 1210	2.54 ± 0.11
**SOCAL**	0	102.1 ± 1.2	–5.76 ± 0.06	–4.10 ± 0.02	7806 ± 930	2.06 ± 0.06
	150	95.5 ± 2.8	–7.41 ± 0.10	–4.09 ± 0.04	9692 ± 1160	2.17 ± 0.09
	212	89.8 ± 3.6	–7.68 ± 0.08	–4.34 ± 0.03	9779 ± 1170	2.06 ± 0.10
	260	83.3 ± 2.8	–7.43 ± 0.06	–3.89 ± 0.03	10323 ± 1230	1.78 ± 0.07
	300	79.4 ± 3.6	–9.88 ± 0.08	–4.27 ± 0.04	10712 ± 1280	2.23 ± 0.11
	357	70.3 ± 2.2	–10.29 ± 0.30	–4.83 ± 0.07	8560 ± 1020	2.00 ± 0.10
	406	67.7 ± 2.4	–13.43 ± 0.26	–5.72 ± 0.04	8810 ± 1050	2.44 ± 0.12
	450	67.2 ± 3.0	–13.57 ± 0.29	–4.54 ± 0.08	10252 ± 1220	2.35 ± 0.13

### Dependence of Initial Contact Angle on the Voltage

**Figure 3:**
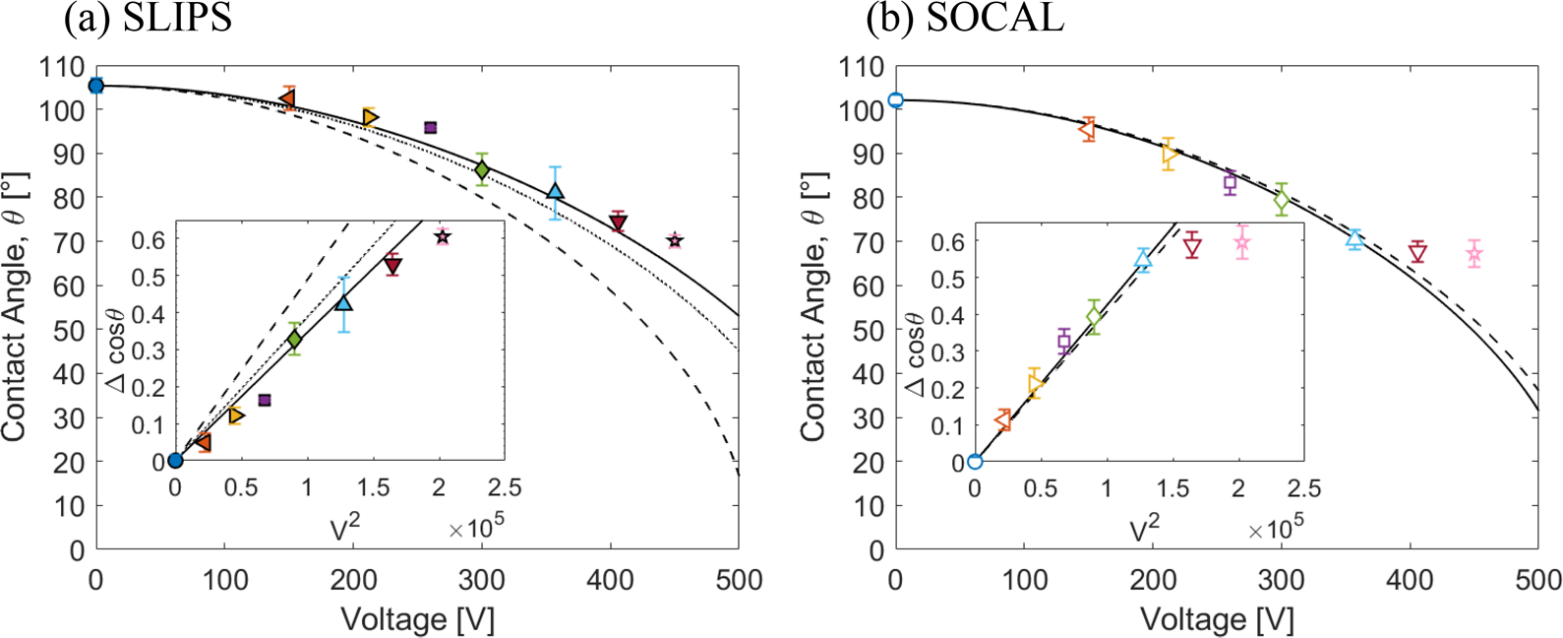
Cosine of average contact angle during evaporation (constant contact angle regime) as a function of voltage: (a) SLIPS and (b) SOCAL. Inset shows Δcos θ as a function of *V*^2^_rms_ fit (solid line) before saturation (voltage rms < 400 V). The dashed and dotted lines for SLIPS are predictions from theory assuming the droplet is cloaked and not cloaked in oil, respectively. The dashed lines for SOCAL are predictions from theory.

We now consider the consistency of the observed voltage-selected contact angles with expectations from the theory of electrowetting. In the absence of contact line pinning, the initial contact angle without an applied voltage is assumed to be given by Young’s law. The effect of applying a voltage and charging a dielectric using the contact area of a droplet as one electrode is to introduce a capacitive energy in addition to interfacial energies. This causes a voltage dependent contact angle, θ(*V*), described by the Young-Lippmann equation
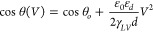
15where θ_0_ = θ(*V* = 0) is the initial contact angle prior to the application of a voltage, ε_0_ is the permittivity of free space, ε_*d*_ is the relative permittivity of the dielectric, and *d* is the dielectric thickness. We therefore expect a quadratic power law dependence of cos θ on the voltage, and this is confirmed for both the SLIP and SOCAL surfaces by Figure 3. The insets in Figure 3 show a linear plot of the Δcos θ with *V*^2^, and the saturation effect of wetting is clearly visible for the SOCAL surface.

To compare quantitively to the theoretical expectations from eq 15, we first consider the slippery SOCAL layer. This has a sufficiently small thickness (ca. 4 nm) to be a negligible correction to the dielectric thickness due to the 100 μm thick glass substrate, and the covalently attached PDMS chains mean there is no cloaking of the droplet-vapor interface. The glass has a manufacturer-stated relative permittivity ε_*glass*_ = 6.7; therefore, using a surface tension for water of γ_*LV*_ = 72.8 mN m^–1^ gives ε_*o*_ε_*d*_/2γ_*LV*_*d* = 4.07 × 10^–6^ V^–2^. This can be compared to the slope Δcos θ/*V*^2^ in the inset in Figure 3b for the experimental data for SOCAL. The experimental result of (4.20 ± 0.10) × 10^–6^ V^–2^ using data below the contact angle saturation voltage of 400 V is in excellent agreement with the theoretical value.

We now consider the consistency theory between the theory and the experimental results for droplets on SLIPS. In this case, we regard the glass substrate and the SLIPS layer as a series capacitive combination of the glass with the SLIPS layer so that the relative dielectric thickness is (*d*/ε_*r*_)_*total*_ = (*d*/ε_*r*_)_*glass*_ + (*d*/ε_*r*_)_*SLIPS*_. The major contribution to the capacitance is therefore from the glass substrate and this gives an order of magnitude estimate consistent with the experimental data. To estimate the small correction due to the SLIPS, we use the thickness of the porous Glaco layer, ∼2 μm, infused with silicone oil with excess oil removed through rinsing, as the thickness of the SLIPS later. Moreover, since the relative permittivity of the silica nanoparticles (ε_*r*_ = 2.5–3.5) and oil (ε_*r*_ = 2.68) in the SLIPS layer are similar and the layer is a small correction, we can approximate it to a uniform dielectric layer with ε_*r*_ ∼ 2.68. This provides an estimate of (*d*/ε_*r*_)_*SLIPS*_ = 6.38 × 10^4^ m. In addition to these dielectric considerations, we also expect the droplet-vapor interface to be cloaked so that an effective interfacial tension should be used in eq 15 replacing γ_*LV*_ by γ_*Eff*_= γ_*WO*_+γ_*OA*_, where γ_*WO*_ and γ_*OA*_ are the water-oil and oil-air interfacial tensions, respectively. Using the interfacial tension data from Banpurkar et al.,^43^ the oil–water interfacial is estimated at γ_*OW*_ = 38 mN m^–1^, and from the data of McHale et al.,^20^ the oil-air interfacial tension is γ_*OA*_ = 19.8 mN m^–1^ giving an effective interfacial tension of γ_*Eff*_ = 57.8 mN m^–1^. Including this cloaking effect gives ε_*o*_ε_*d*_/2γ_*Eff*_*d* = 4.89 × 10^–6^ V^–2^ and thus overestimates the contact angle changes compared to the experimental data (dashed line compared to symbols in Figure 3a). However, assuming that oil does not cloak the droplet-air interface gives ε_*o*_ε_*d*_/2γ_*LV*_*d* = 3.88 × 10^–6^ V^–2^, where γ_*LV*_ = 72.8 mN m^–1^, and this is closer to the fit to the data which is ε_*o*_ε_*d*_/2γ_*LV*_*d*=(3.47 ± 0.11) × 10^–6^ V^–2^ (dotted lines compared to solid lines in Figure 3a). This is contrary to expectations on the state expected from the analysis by Smith et al. (i.e., their A3-W3 state) and therefore suggests an additional effect from the electric field.^44^ To fit the curve using eq 15 and an oil-cloaked droplet-air, we would require a significantly smaller value of the relative permittivity (∼75%) than the manufacturer-provided value and/or a significantly thicker (∼40%) glass substrate.

### Droplet Lifetime Dependence on Contact Angle

**Figure 4:**
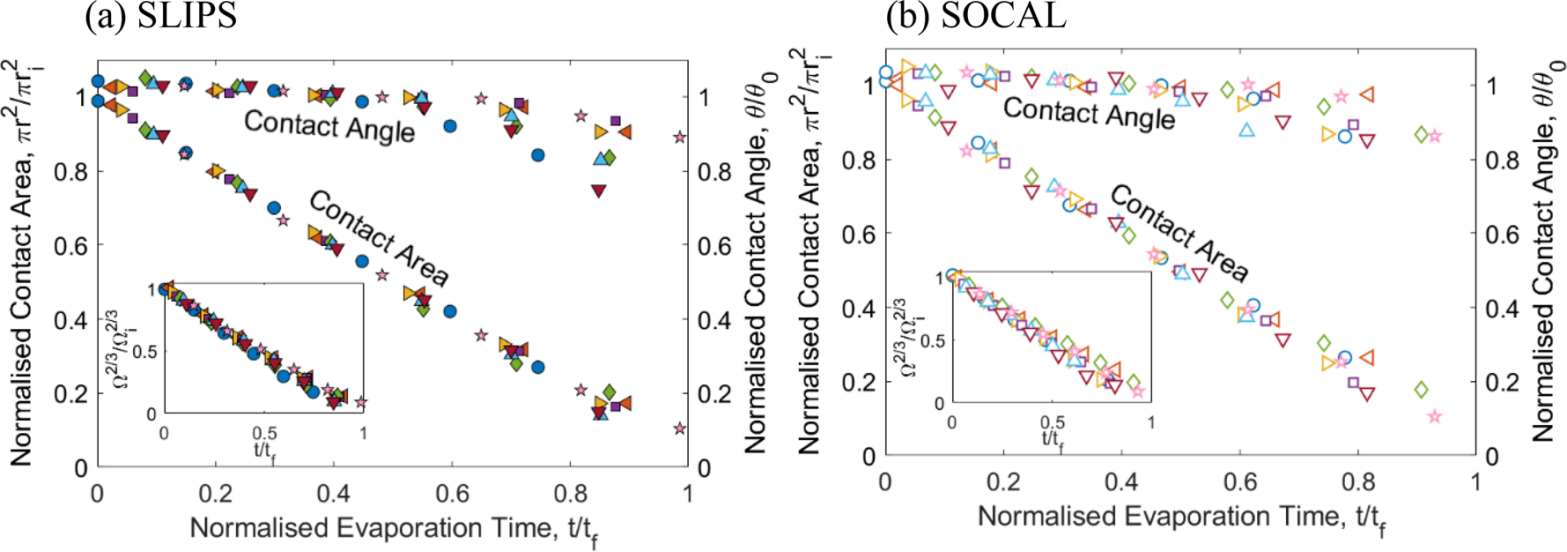
Scaling of evaporation measurements with droplet lifetime. Normalized contact area, π*r*^2^/π*r*_*i*_^2^ as a function of normalized time, *t*/*t*_*f*_ and normalized contact angle, θ/θ_c_ as a function of normalized time *t*/*t*_*f*_. Insets: normalized volume Ω^2/3^/Ω_*i*_^2/3^ as a function of normalized time, *t*/*t*_*f*_. Data presented is every 50th data point for clarity of presentation.

We now consider the extent to which the lifetime of an evaporating droplet, *t*_*f*_, depends on its initial contact angle. From the fits to the contact area for each evaporating droplet sequence, the lifetime was determined (Figure 2 inset), and these values are given in Table 1 for each surface. For scaling dependence, Figure 4 shows the contact area normalized by the initial contact area (using the intercepts in the insets in Figure 2) as a function of time normalized by total evaporation time for droplets on each surface. The secondary *y*-axis in each figure shows the contact angle normalized by the constant contact angle. A similar collapse of data onto a single scaling curve can be observed for the 2/3 power law for the drop volume, and this illustrates the good agreement with the power law on these slippery surfaces (insets in Figure 4).

Equation 13 shows that the lifetime is a separable product of three functions involving the constant contact angle, θ_*c*_, the initial droplet volume, Ω_*i*_, and the parameter λ which incorporates the density, ρ, difference in vapor concentration, Δ*c*, and diffusion coefficient, *D*, i.e.,
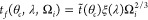
16where the contact angle dependent factor is
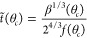
17and various other dependencies have been grouped together as
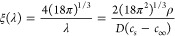
18

**Figure 5:**
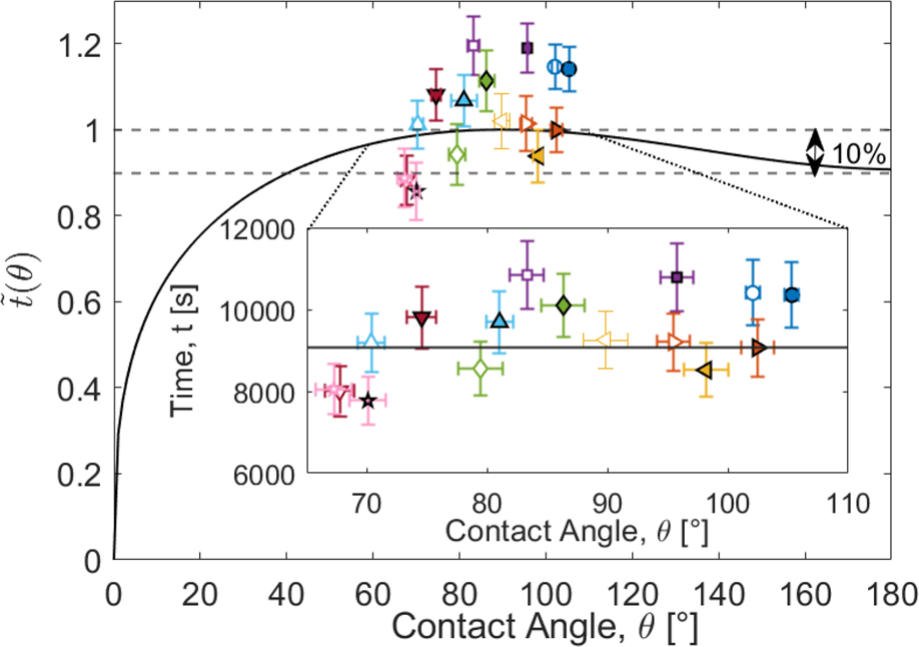
Drop lifetime contact angle dependence factor, *t̃*(*θ)* (Solid line theory, solid symbols are experimental data for SLIPS and empty symbols are experimental data for SOCAL surfaces). Inset: expanded view of the contact angle range 67°–105° (i.e., cos θ = 0.39 to −0.26) plotted with absolute time as the vertical axis (eq 16).

According to Stauber et al., the droplet lifetime in the constant contact angle mode of evaporation has a maximum at θ = 90°.^36^ This is illustrated by the solid curve in Figure 5 showing a plot of eq 17 over the full contact angle range, from a film with θ = 0°, to a spherical sessile droplet with θ = 180° using the Picknett and Bexon polynomial interpolation (eq 7) for *f*(θ) in eq 17. To understand the contact angle dependence of the total evaporation time for surfaces with contact angles close to the maximum (i.e., θ = 90°), we approximate eq 17 to quadratic order around θ = 90° using Stauber et al.’s formula for *f*(θ). This gives a quadratic expansion approximation around the maximum of
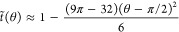
19where θ is in radians. One can obtain the same result by numerically fitting a second order polynomial to eq 17 using the explicit expression *f*_*PB*_(θ) provided by Picknett and Bexon. From the full plot in Figure 5, we note the contact angle dependence is predicted to be relatively insensitive to the precise value of θ and remains within 10% of the maximum value over the contact angle range from 40° to 180°. In experiments using smooth surfaces, i.e., not superhydrophobic, the maximum achievable contact angles with surface chemistry is ∼115° rather than the parameter maximum of 180° in the theory. We have therefore provided an inset in Figure 5 showing the more limited range bounded by the lower limit due to contact angle saturation in electrowetting (∼67°) and the maximum achievable contact angle (105°) on our smooth surfaces; this covers a wide range of cos θ from 0.39 to −0.26.

To analyze the contact angle dependence of the experimentally determined lifetimes, we assume a droplet had an initial volume of (8.0 ± 0.1) μL and a temperature of (22 ± 2 °C) and was evaporated in air with a relative humidity of (70 ± 1%) which gives a value of ξ(λ)Ω_*i*_^2/3^ = 9070 ± 990. The values of droplet lifetime from Table 1 scaled down by this value are plotted in Figure 5 for comparison to the theory with the absolute lifetimes shown in the inset; the average value for *t*_*f*_ from Table 1 is (9330 ± 1000) s. The solid symbols show the data from the SLIP surface and the empty symbols show the data from the SOCAL surface. This data covers a contact angle range from hydrophilic (lowest value θ_c_ = 67°) to hydrophobic 106° (highest value) and shows a scatter around an average value without an obvious contact angle trend. The data appears to lie slightly above eq 17 suggesting a slight systematic error in the value of ξ(λ)Ω_*i*_^2/3^ used in the analysis.

From our results, we conclude that in the constant contact angle mode of evaporation and for constant contact angles above 40°, drop lifetimes can be predicted within a 10% tolerance range without precise knowledge of the exact value of the contact angle by using eq 16 with *t̃* ∼ 1, i.e., drop lifetimes have a weak dependence on the contact angle for a broad range of constant contact angles above 40°. Improved estimates could also be made by calibrating experimentally over a specific contact angle range to use a value of *t̃* slightly below unity, i.e.,
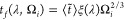
20where ⟨*t̃*⟩ is an experimentally determined calibration constant (with a value close to unity) over the relevant contact angle range, which should be above 40°. It should also be possible to decide a desired tolerance on the droplet lifetime and from that determine what range of contact angles needs to be achieved. In practical applications where drying is important, knowledge of initial droplet volume, the liquid density, the temperature, and relative humidity (or diffusion coefficient and difference in saturation and ambient vapor concentration) should be sufficient to predict drying time providing the surface allows a mobile contact line without contact line pinning and the contact angle is above ∼40°. These results also show that the initial droplet contact area on a slippery surface can be selected when the contact angle is above ∼40° without significantly changing the overall droplet evaporation time.

## Conclusion

Our results show that control of constant contact angle mode evaporation over a wide range of receding contact angles, from hydrophilic to hydrophobic, of droplets on pinning free SOCAL and SLIP surfaces can be achieved by using electrowetting. The results are consistent with the model of diffusion-controlled evaporation of sessile droplets and can be used to estimate the diffusion coefficient. The contact angle-voltage relationship is in excellent agreement with the Young-Lippmann equation for electrowetting of droplets on both types of slippery surfaces. We have also observed that over a range of contact angles above 67° on these surfaces, droplet evaporation times are relatively insensitive to the precise value of the contact angle. Thus, a desired tolerance on droplet lifetime can be used to determine what contact angle range and accuracy is required. This may have practical application in processes involving evaporation, such as inkjet printing, where consistent drying times would then depend mainly on the liquid density, control of droplet deposition volume, and environmental factors, such as temperature and relative humidity.
